# The Machinery of Authoritarian Care: Dramatising Breast Cancer Treatment in 1970s Britain

**DOI:** 10.1093/shm/hku003

**Published:** 2014-04-11

**Authors:** Elizabeth Toon

**Keywords:** breast cancer, patient experience, medicine and the media, doctor–patient relationships, the National Health Service

## Abstract

This article examines the professional and public response to the television play *Through the Night*, which aired on BBC1 in December 1975. One of the first British mass media portrayals of a woman's experience being treated for breast cancer, this play attracted a large audience and considerable attention from both critics and everyday viewers. My analysis of the play draws on sources documenting expert responses to the play in its production stages, as well as critics' and viewers' responses to what the play said about breast cancer treatment in particular, and about Britons' experiences of medical institutions more broadly. Together, I argue, these sources help us see how *Through the Night*'s critique of what one expert called ‘the machinery of authoritarian care’ reverberated with and supported the efforts of professionals anxious to improve patient experience, and how it crystallised the concerns of activists and everyday viewers.

In early December 1975, television channel BBC1 presented *Through the Night*, the story of a young working-class woman with breast cancer and her encounters with the medical establishment. Although at least one prominent reviewer confessed that he ‘lacked the nerve to face’ the programme, an estimated eleven million viewers did tune in that evening.^1^ What they saw was one of the first mainstream British mass media accounts depicting the experience of breast cancer treatment, following one woman's institutional and emotional trajectory from initial examination through treatment to the beginnings of recovery.^2^ Granted, this woman—named Christine Potts—was a fictional character, played by the up-and-coming actress Alison Steadman in a drama by playwright Trevor Griffiths. But even if Christine was a fictional creation, attentive viewers would see that her experiences were based in real life, when the play's credits revealed that the script was ‘based on a diary kept by Jan Griffiths’. What's more, those who watched *Through the Night* would find that this teleplay did something fairly radical compared to most medical dramas of the time: it explicitly encouraged viewers to see cancer treatment—and medical care in general—through the patient's eyes.

Media scholars have noted *Through the Night*'s unusually forthright depiction of mastectomy, and have also commented on the fact that the teleplay received an especially enthusiastic and extensive public response.^3^ But as historians of British medicine will recognise, the teleplay's frankness marked an especially significant change from previous media representations and discussions of cancer in the UK. Before the late 1960s, the nation's newspapers, magazines, and radio and television programmes generally presented cancer as a diffuse scourge to be conquered by science, charity and the state, and their discussions of cancer usually highlighted biomedical research news or announced new equipment and facilities acquired through private giving or government action. Nor did the British media discuss specific cancer symptoms, treatments, outcomes or experiences in much detail. When Britons in the public eye suffered from and died of cancer, few mass media accounts precisely identified the cause of death. Savvy readers might catch hints between the lines of obituaries, and journalists and memorialists might later identify cancer generally as the illness responsible, but they still rarely mentioned which specific form of cancer was involved.^4^ Indeed, even those who made it their business to deal with cancer—medical and public health authorities—were restrained in their public discussions of the disease, especially when their efforts were compared to highly visible anti-cancer campaigns in the United States and elsewhere.^5^ As Ornella Moscucci has shown, some interwar medical advocates and local government authorities in Britain had mounted cancer education campaigns, usually focused on breast and cervical cancer.^6^ But in the decade immediately following the Second World War, British cancer education's momentum slowed when faced with GP resistance and policy-makers' preference for improving service provision first.^7^ Even when the mass media presented positive messages about cancer treatment, many medical commentators objected to televised discussion of the subject.^8^ This persisted until the late 1950s, when the link between smoking and lung cancer encouraged policy-makers and health educators to make anti-smoking work a priority; likewise, newspapers and magazines in the early 1960s began to trumpet early detection of cervical cancer through smears, feeding a vocal campaign by women's groups to make screening a national priority.^9^ However, even these expanded media discussions and educational efforts focused mostly on the advantages of prevention and early detection, and had little to say about cancer treatment or everyday people's experience of it.

By the late 1960s, this situation had begun to change. In 1968, novelist Penelope Mortimer's *My Friend Says It's Bulletproof* featured a main character returning to work after a mastectomy, and that same year education journalist Caroline Nicholson briefly discussed her own experience with breast cancer in the *Guardian*.^10^ Finally, in 1973 breast cancer treatment took centre stage in British magazines—or at least those aimed at women. That March, the self-consciously modern women's monthly *Nova* featured a lengthy investigative piece by Nicholson, who interwove her own and other women's personal experiences into her review of treatment trends at home and abroad.^11^ The next month leading weekly *Woman's Own* took the subject to a much broader audience, devoting several pages to a personal narrative by the American child actress-turned-diplomat Shirley Temple Black, reprinted from the US woman's magazine *McCalls*.^12^ These relatively explicit discussions of breast cancer treatment appeared in an context where, as Alex Mold has shown, organisations claiming to speak for patients urged Britons receiving medical care to be aware of their rights in health care settings.^13^ They also urged their female readership to get more involved in their own health care, by seeking out more information, and if diagnosed, to be prepared to take an active role in determining how their breast cancers would be treated.

Coming on the heels of these discussions, *Through The Night* (hereafter *TTN*) opened the subject of breast cancer to broad public discussion, by moving the subject from the pages of women's magazines to a prime-time slot on the national broadcaster. The teleplay's airing, and the media debates that followed, thus illustrate a crucial shift in the history of cancer in Britain, a shift that made sufferers' experiences of cancer and its treatment a central element in representations of the disease. But to fully understand what this play can tell us about late twentieth-century British medical care, we need to move beyond asking what *TTN* said about the experience of cancer treatment, and also see how the multiple messages it appears to have communicated to diverse audiences were both shaped and interpreted. To do that, this article begins by examining *TTN*'s origins in Jan and Trevor Griffiths's own experiences, and by outlining how the play portrayed the fictional Christine Potts's experience. It then explores the negotiations that shaped the telling of Christine's story on British television, when the BBC had the play's text vetted by medical experts, cancer educators, and women's health advocates. By looking at why and how sympathetic health professionals and activists supported *TTN*'s portrayal of patient's experience, I show how some 1970s medical professionals hoped to refashion both the practice of cancer treatment and the larger institutional structures that delivered it.

*TTN* also triggered public discussion of doctor–patient relationships in general, at a time when those relationships were increasingly fraught. Unusually a broad array of media sources documenting elite and popular responses to the play and its subject matter is available, ranging from broadsheet reviews to tabloid correspondence to audience surveys. Although these responses are of course refracted through the concerns and commitments of the media that collected them, they nonetheless allow historians of medicine insight into how and why Christine's story reverberated with the everyday women and men who viewed it. This study thus complements and extends recent analyses of efforts by organisations, activists, and bioethicists to create a patient (and subject) ‘voice’ in 1960s and 1970s British medicine.^14^ Popular responses to *TTN*, I conclude, reveal how deeply the play's critique of what one expert called ‘the machinery of authoritarian care’ resonated beyond concerns about breast cancer treatment, crystallising existing professional debates and public worries about the British health care system.^15^ By examining how Britons reacted to Christine's story, who identified with it, and why they found it realistic and compelling, we can get a better picture of what everyday patients felt they deserved from the health care system, and how they thought that system needed to change in order to deliver it.

## The play: Personal story and political critique

When Jan Griffiths entered hospital, she may not have expected her experience to be fictionalised for an audience of millions. A social worker from a politically active family, she had married playwright Trevor Griffiths in the 1960s, although they would later separate. (She died not of breast cancer, but in a 1977 plane crash.) She found a lump in her breast in 1972, at age 27, but when she consulted her GP about it the GP judged it unlikely to be dangerous, given Griffiths's age, and referred her onwards for biopsy only because the lump was increasing in size. After a six-week wait, Griffiths was admitted to hospital in Leeds, and when her surgeon performed the operation and found an aggressive, fast-growing form of breast cancer, a mastectomy was performed.^16^

Her husband was at the same time casting around for his next project. The son of a Mancunian factory worker, Trevor Griffiths had been part of the first generation to benefit from the Education Act of 1944, and studied at Manchester University and became involved with leftist politics. He then went on to work first at a local college and then as a teacher at Stockport Technical College, while beginning to write.^17^ From the first, his work was explicitly political, often centring on issues of class and institutional authority. Some plays used history to address the Left's past and future, while others examined the ‘accidents of education’ that could produce very different life trajectories.^18^ By the early 1970s, Griffiths's work had attracted considerable interest: he had, for instance, been commissioned by the National Theatre Company's Kenneth Tynan to write *The Party*, which featured Sir Laurence Olivier in his last stage role. Griffiths had also begun writing for television as well as the stage, optimistic about the ways television drama (and mass culture generally) could lead to political change, an approach termed ‘strategic penetration’.^19^ Before his wife's diagnosis and after the BBC's *Play for Today* strand had presented his *All Good Men*, Griffiths had been commissioned by Granada to write a television play. Having read a piece in *New Society* about a pioneering youth remand centre, he had hoped to use that as his basis for a new play, but found it difficult to get access to the facility.^20^

Then his wife Jan entered hospital. Griffiths visited frequently and, as he remembers, soon ‘saw the play quite clearly in [his] mind,’ taking notes on the people and surroundings in a writer's attempt to make sense of a difficult situation:

I sat around and just did what I do which is watch, and … everybody who writes creatively like this has two collateral stances, one is deep emotion and involvement and the other one is standing outside and watching.^21^

Griffiths also suggested that Jan keep her own diary of her experiences.^22^ In the finished play, the background and personality of the central character, working-class housewife Christine Potts, differed considerably those of Jan Griffiths; likewise, the character of Joe Potts, Christine's husband, was in no way a direct stand-in for Trevor Griffiths himself. But the observations made by both of the Griffiths during Jan's time in hospital, together with her experiences, formed the raw material that was then transformed into the script of *TTN*.^23^

The play opens with Christine, a northern working-class wife and mother in her late 20s, being examined as an outpatient by consultant Mr Staunton, house surgeon Dr Seal (a woman) and junior doctor Dr Pearce. Staunton and Seal palpate Christine's breast as a matter of course, but the slightly scruffy, regionally-accented Dr Pearce (played by Jack Shepard) marks himself as a different kind of doctor from the start, by asking Christine's permission to examine her breast. Staunton confers with Seal and Pearce, unwilling to mention the word ‘cancer’ in front of Christine, and then explains to her with what stage directions refer to as a ‘practised smile’ that he will ‘arrange for you to come in so that we can do a tiny operation and find out what it is’.^24^ In the next scene, set several weeks later, Christine enters the hospital, taking a bed in a large open ward. The scenes that follow allow the viewer/reader to see a day in hospital life through Christine's eyes: an elderly woman patient moans and cries, while overworked nurses fumble another patient's blood change and spill it.

When Seal, the female surgeon described as having ‘cold hands’, brings a consent form to Christine the day before her operation, she quickly explains that the forms are for a biopsy and hurries her patient to sign: ‘We'll cut a piece of the lump and, er, do some tests on it … and the tests will tell us what to do next, if anything. Sign there, if you will.’^25^ But just before the operation, and after the anaesthetist has given Christine the pre-anaesthetic in preparation for surgery, Seal returns with another, modified consent form. ‘It's nothing to worry about,’ Seal briskly tells Christine. ‘Mr Staunton thought it might be as well if you signed an open consent form too. Just in case.’ The televised play carefully follows the stage directions in the written version, with the camera scanning the consent form in Seal's hand, and ‘Casually pick[ing] out key words … “Frozen section (biopsy)? Proceed? Mastectomy”’. Seal then says, soothingly, ‘Then if there were anything, well, nasty he could deal with it on the spot, instead of having to send you home and call you back again.’^26^ Indeed, in the operating room, Staunton performs an open biopsy, and then he and his staff wait, with Christine still under anaesthesia, for a verdict on the tissue sample from the pathologist. Once the surgical team receives the biopsy report, Staunton proceeds with a mastectomy; this combined biopsy-mastectomy, known as the ‘one-step’, remained common practice in most Western countries at the time.^27^

When Christine wakes up back on the ward, she realises what's happened and cries out ‘What have they done to me?’^28^ The next day her husband Joe visits, and because Christine has been unable to determine what is happening to her, she asks him to find out. Joe Potts speaks to the head ward sister (although the viewer does not hear what is said), and returns to Christine's bedside to explain that because the doctors found ‘an infection’ they did a full operation. He then tries to reassure her that all will be well, although Christine is unconvinced. Her confusion and anger are only exacerbated when Mr Staunton comes to do rounds, with his juniors and the head sister trailing him around the ward. In the televised play, the viewer sees the doctors and the head sister from Christine's viewpoint: they cluster at the foot of Christine's bed and talk quietly to each other, and both Christine and the viewer are allowed to catch only a few of the words and phrases Staunton, Seal and Pearce say before they move on to the next bed.^29^ The next morning, as Christine opens her tabloid newspaper, she is greeted with the usual sight of the *Sun*'s Page Three ‘dolly’, a half page photo of a glamour model naked from the waist up, displaying her large breasts.^30^ Dr Seal arrives, and after explaining that Staunton had ‘done everything that was needed’ tells Christine that the team will want to talk to her when tests are back: ‘Nothing to concern yourself over, they're just routine tests we carry out in cases like yours.’^31^

But although Christine's husband and mother have tried to convince her that the hospital staff have acted for the best, the quiet young woman finally revolts. Late that night, she locks herself in one of the toilet stalls. When the ward nurses cannot persuade her to come out, they enlist the young houseman Pearce, worse for wear from a night out, to help. After making Christine laugh with a Humphrey Bogart impression, he convinces her to come out of the toilets, and he wheels her off to the staff quarters to make cocoa and talk. In a small but increasingly confident voice, Christine says that:

Nobody says anything. They treat you as if you were already dead. The specialist, he never even looked at me, let alone spoke. (*Long pause.*) I know it were serious. I'm not a child. You don't cut a thing like that off for nothing. (*Long pause.*) I don't want … fobbing off.^32^

Having poured the cocoa, Pearce finally answers Christine's questions. In careful but clear language, he tells her she was diagnosed with breast cancer, that despite the operation it may have spread, and that she may need radiotherapy.

It's … hard to explain why you haven't been told all this, why we go on talking about this ‘infection’ and ‘nasty tissue’ … I mean, there are a thousand reasons, most of them decent and honourable …

Mainly, I think, it's because we have lost all idea of you as a whole, human being, with a past, a personality, dependents, needs, hopes, wishes. Our power is strongest when you are dependent upon it. We invite you to behave as the sum of your symptoms. And on the whole you are pleased to oblige.^33^

He later quotes Hippocrates at length in an apology for the failings of modern medicine:

‘… For whoever does not reach the capacity of the common people and fails to make them listen to him, misses his mark.’ Well, we're all missing the mark, Mrs Potts. And we need to be told. Not just doctors and nurses, but administrators and office men and boards of management and civil servants and politicians and the whole dank crew that sail this miserable craft through the night.^34^

Though surprised at learning she has been operated on for breast cancer, Christine is calm and thoughtful, almost satisfied to have finally been told what her condition is. The next day, her change in demeanour is clear: when the young South Asian nurse Chatterjee comes to change her dressing, Christine is not only able to look at her scar but jokes ‘What did he do it with, a bottle?’^35^ The play concludes with working-class housewife Christine joining a hip young student and a troublesome but good-hearted widow for some contraband gin and a giggle. Seated on a bed in the corner of the ward, they express a boozy, earthy female camaraderie in the face of the cold, sometimes incompetent, but mostly well-meant medical bureaucracy of the NHS. Toasting behind the bedcurtains, they each first say ‘sod it’, and then whisper in unison—in the published version of the play—‘fuck it’.^36^

## Negotiating critique: Producing and presenting *Through the Night*

Trevor Griffiths offered his script to Granada Television, the well-regarded production company responsible for much of the broadcasting on the nation's third television channel, ITV, as well as some highly-rated productions that featured on the two BBC channels. But Granada executives, worried about the effort it would take to get it past their board, passed on the play. Ann Scott, a producer Griffiths had worked with previously, convinced the *Play for Today* group at the BBC to take it on, and with Scott as producer and Michael Lindsay Hogg as director, the play began to move towards production. But the BBC almost immediately demanded a number of changes to the play, starting with its working title *Maiming of the Parts*, a play on the poem *Naming of the Parts* by Henry Reed. After considering a number of alternatives, Griffiths took writer Kenneth Tynan's suggestion and opted for *Through the Night*.^37^ BBC1 controller Bryan Cowgill also objected to the words ‘fuck it’ at the end of the play. Griffiths argued strongly for keeping the line, maintaining that the ‘tough, ironic toast … carries the play's meanings, [and] is the right and real and proper ending’. To *not* say ‘fuck it’, he insisted, infantilised the audience. Cowgill responded that despite the word's likely appropriateness and power in this play, ‘fuck’ was simply not permitted.^38^

But even beyond these script changes, producing a play on a medical theme meant that *TTN*'s production team had to deal with considerable scrutiny. Not only was the play about cancer, and to be shown on the national broadcaster, but it also came at a moment of great concern about how medicine and medical topics were to be handled on television. In the past, the BBC had faced significant criticism when the British Medical Association objected to episodes on cancer treatment in the 1958 medical documentary series *Your Life in Their Hands* (*YLITH*)*.* When a second series of *YLITH* was commissioned in 1961, BBC producers hoped to avoid a repeat of the situation by seeking the explicit endorsement of leading medical figures in advance. Meanwhile, the BBC itself assigned an executive to liaise with producers, the medical profession, and the Ministry of Health.^39^ Such collaboration grew more frequent in the 1960s, as Ayesha Nathoo, Kelly Loughlin and Anne Karpf have shown. This was especially true for programmes understood to be complimentary of medicine, such as ITV's *Emergency Ward 10* (aired 1957–1967), which the Ministry of Health hoped would make hospitals less frightening to the public.^40^ But by the 1970s, televised critiques of the medical profession, medical institutions and the National Health Service had begun to feature more frequently on television, in part thanks to current affairs programmes. One especially visible critique, an episode of BBC's *Horizon* challenging the routine induction of labour in childbirth, aired in early 1975. Even though the main critical voice in the documentary was that of a doctor, the London Hospital's Peter Huntingford, the airing of his then-‘maverick’ position spurred a *British Medical Journal* editorial urging doctors ‘to be more than ever cautious about taking part in these programmes and in opening up their units and their patients to the film cameras’.^41^

Given this incident earlier in the year, it is not at all surprising that medical and health experts were asked to evaluate the *TTN* script, presumably to catch any errors but also to make the BBC less nervous about potential criticism once the programme aired.^42^ *TTN*'s producer Ann Scott pulled together a list of reviewers from suggestions made by both Griffiths and the BBC, as well as by charities such as the Marie Curie Memorial Foundation.^43^ Among those who provided evaluations were two highly respected cancer educators: John Wakefield, who led the Christie Hospital, Manchester's pioneering Social Research Department, and the Health Education Council's A. Dalzell-Ward. Two psychiatrists also commented on the script: Dr Peter Maguire from the University Hospital of South Manchester, who had recently published research on how women and their husbands dealt with mastectomy, and the Middlesex's Dr Edward Chesser, who had also recently investigated psychosocial aspects of breast cancer. A young surgery lecturer, J. H. Wheeler, read over the script on behalf of the Junior Hospital Doctors Association. Finally, the reviewers included two women who were not medical professionals: Jill Rakusen, an activist and educator working at that point with the group AWARE (Action for Women's Advice Research and Education) who later became the co-author of the UK version of *Our Bodies Ourselves*; and Betty Westgate, the founder of the Mastectomy Association.^44^

All these commentators commended the script, agreeing that while *TTN* depicted many uncomplimentary examples of careless practice and bureaucratic mistakes, such practices and mistakes were, sadly, accurate depictions of the realities of hospital care.^45^ Nevertheless, they urged the correction of some minor errors, noting for instance that a junior nurse would not write notes in a patient's chart. Still other minor changes grew out of the educators' and doctors' worries about the unintended messages viewers would take away from the play. For instance, the health educator Wakefield urged Griffiths to change Pearce's description of radiotherapy, from a ‘blast’ (which he argued might frighten some patients); he also noted that Pearce should describe radiotherapy as a potential treatment rather than an expected one, as it might make viewers who had not received radiotherapy worry that their own treatment had been lacking. Given that British breast cancer specialists at that time disagreed whether X-ray therapy was a necessary adjuvant to mastectomy, changing the way Pearce described radiotherapy would also allow the script to avoid falling foul of medical debates around this ‘unresolved’ question.^46^

One of the aspects of the script receiving most scrutiny, though, was the scene where Christine is asked to sign a second, expanded consent form after already having been administered pre-anaesthetic. All those commenting singled this scene out, most arguing that this series of events was unlikely. The surgeon Wheeler, for instance, pointed out that extracting consent after drugs had been administered opened a hospital up to legal action from the patient, and that doctors were aware that they were legally bound to make sure patients understood consent forms.^47^ Chesser, the psychiatrist, believed it was possible a patient might be asked to fill in a second consent form, but also noted that these forms ended with the sentence ‘the nature of this treatment has been explained to me’. He argued that this meant doctors knew they were obliged, legally, to explain any additional or changed treatment plans, in more detail than Seal does in the play.^48^ Even health educator and activist Rakusen suggested altering the scene so that Christine was asked to sign the altered consent form before being given pre-anaesthetic, as was much more likely to occur. Rakusen was careful to note, however, that she believed such a change in the sequence would be ‘in the interest of clarity, not in the interest of the medical profession or anybody else’.^49^ But on this point Griffiths and the production team held firm, because (as the playwright has discussed publically since), the hurried presentation of the modified consent form *after* the administration of pre-anaesthetic is what had actually happened to Jan Griffiths.^50^

The focus on whether these particular events and characterisations were likely to occur or even possible led many of those considering the play to try to answer a larger question, posed most succinctly by Chesser: ‘Could this happen in real life and if so, how representative is it?’^51^ Could a breast cancer patient be deprived of information, and thus the ability to make decisions about her treatment, in the way Christine was? As people familiar with medical settings and with professional practices, the commentators realised that they were being asked not just to catch errors in presentation, but to consider the truthfulness of the play as a claim about medical care and patient experience, both individual and collective. So while Chesser, Maguire, Dalzell-Ward, Wakefield, Rakusen and Wheeler had different opinions as to how *likely* the exact series of events depicted in *TTN* were to occur, all agreed that the play was essentially truthful in capturing a breast cancer patient's experience, in an especially dramatic but nevertheless *real* way. Chesser, for instance, concluded that the play's characters might be exaggerated and stereotypical and that Christine's time in hospital was ‘more horrific than is usually the case’. But, having just conducted a study of doctor–patient communication in breast cancer treatment, he found the play's broad theme ‘realistic’.^52^ Certainly, Christine's experience of doctor–patient communication was in keeping with the findings of Chesser's study (presented in February 1975 but, coincidentally, published the same week *TTN* aired). Chesser and his colleagues had interviewed patients admitted to London's Middlesex Hospital for breast surgery, and found that at initial consultation ‘Most patients were given no information about treatment or were only told vaguely about “a removal”.’ Once in hospital, these women were likely to get more specific information about their potential diagnoses from housemen than they were from consultants and registrars.^53^ Most important, Chesser's study found that good doctor–patient communication—by which he meant the sharing of specific and definite information about diagnosis and prognosis—meant fewer problems after treatment, with women claiming to have received such information scoring lower for anxiety and depression on psychiatric scales.^54^

The other psychiatrist asked to comment on the draft script of *TTN*, Peter Maguire, enthusiastically endorsed it as ‘an impressively accurate picture of a patient's experience’. What's more, he argued that it was important to broadcast this ‘very valuable account of what the average patient experience[d]’, though it was uncomplimentary of medicine, because it was better to ‘air the problem in this way, rather than to pretend that this is in any way exceptional’.^55^ Indeed, Christine's experience and Griffiths's telling of it was very much in keeping with the findings of the research Maguire and his surgical and nursing colleagues had been doing at the University Hospital of South Manchester. Published in the *Nursing Mirror* and later in the *BMJ*, the work done by Maguire and his colleagues was amongst the earliest British research to consider how counselling and aftercare could improve women's experiences of mastectomy. Certainly the stories his interviewees told indicated that the fictional Christine Potts's story had many real-life analogues. One widow remembered that

At night another doctor asked me to sign a form agreeing to the operation … just a vague possibility of further surgery … but he thought it most unlikely. He inferred it was just a formality … but I couldn't sleep. I hadn't thought I'd need a big operation. No one had told me anything. I'm not an imbecile. I like to know what they are going to do. I have a right to know.^56^

Many of the women Maguire's team had spoken to shared this woman's experience of having been given ‘inadequate information and insufficient opportunity to ask questions or discuss … worries’; some were unhappy that they had been given no time to discuss the surgery with their husbands, while others even ‘alleged that the possibility of mastectomy was glossed over or denied’.^57^ But, as in Christine's case, the women angered by this situation in hospital felt they had few means for expressing their anger or getting more information because, as Maguire wrote,

much of this distress remained undetected by the nursing and medical staff. The reasons for this … included complaints that it was difficult to say how you were feeling when the main aim of the staff seemed to be to jolly you along.^58^

Maguire's later work argued strongly for specialist nurses providing aftercare and counselling to mastectomy patients. There, he and his nursing colleagues would tease apart just as deftly in professional prose the phenomenon Griffiths had previously dissected through drama: ‘a conspiracy of pretence’ where patients were afraid to trouble busy nurses and doctors for information, where nurses and doctors avoided frank disclosure in favour of keeping up spirits, and where ‘both cancer patients and staff conspire[d] to pretend that the patients are coping well, emotionally’.^59^

Interestingly, whether they found Christine's experience as a breast cancer patient to be merely ‘realistic’ or ‘impressively accurate’, none of the experts whose verdicts on *TTN* are available commented on the fact that Christine herself differed from most breast cancer patients in one crucial respect: she was a relatively young woman, in her late 20s. In the 1970s, as now, most women undergoing investigation and treatment for breast cancer tended to be middle-aged and older. Indeed, of three patients featured in *TTN*—the housewife Christine in her late 20s, the early 20s student Anna, and the much older, widowed Mrs Scully—Mrs Scully was the one who in real life would be most likely to be suffering from breast cancer, given the disease's age distribution. The play, though, indicates that Mrs Scully has had abdominal surgery, while Anna and Christine are identified as having been admitted for breast biopsy. Given that Christine's experience was based on that of Jan Griffiths, who was in her late 20s when treated, it is not surprising that the main character of *TTN* would be close to her in age. Nevertheless, even though Christine was meant to be a real patient if not a strictly representative one, her youth and the beauty of the actress, Alison Steadman, portraying her may well have intensified viewers' sensitivity to her tragedy.

Certainly, those asked to evaluate the *TTN* script recognised that they had been asked to consider not just the messages the teleplay sent about mastectomy and breast cancer treatment, but patient experiences of health care generally. Wakefield the cancer educator admired the realism of the play, which he argued resembled Solzhenitsyn's *Cancer Ward* in presenting a necessary portrait of ‘agonizing helplessness, uncertainty and fear … man caught up in the machinery of authoritarian care’.^60^ Even though Wakefield firmly supported airing the play, such truthfulness, he admitted, might make his task as a cancer educator harder, as ‘There is nothing in [the play] to spur others on to behave sensibly.’^61^ Meanwhile, the women's health educator and activist Jill Rakusen took the most head-on approach to the question of how truthful and ‘real’ Christine's experience was, and why that mattered. Leading medical authorities, she suggested, would probably argue that with its poor consent procedures and withholding of information *TTN* depicted bad old practices rather than what happens ‘nowadays’. But, she argued, such authorities, by virtue of simply being authorities rather than patients or everyday staff, did not really know what the patient's experience of their facilities was like. Even so, Rakusen concluded, if *TTN* did depict rare or outdated occurrences, ‘so what! They have happened, they can happen.’ If the play demonstrated that such things *could* happen, the public ‘would be in a better position to make sure that such occurrences do not happen’.^62^ As we shall see, many of the viewers who tuned into *TTN* would concur both with Rakusen's certainty that such things happened, and with her belief that this needed to change.

## Not necessarily typical, but true: Responses to *Through the Night*

*Through the Night* aired for the first time on 2 December 1975. In response to the BBC's concerns about the play's impact on women who had been treated for breast cancer, the channel also aired a discussion on *Tonight*, with Griffiths, a doctor and a psychiatrist, and hosted by presenter Dennis Tuohy, immediately afterwards.^63^ As mentioned previously, the play attracted a comparatively large audience, an estimated eleven million viewers, more than any other production in the *Play for Today* strand since its reintroduction two years previously.^64^ The sheer size of the audience may have been due to publicity and advance reviews for it in a relatively wide range of media outlets: the *Radio Times* that week featured a profile of Griffiths, and on the day it was scheduled to air, television critics from the *Daily Telegraph*, the *Daily Mirror*, the *Sun*, and the *Daily Mail* all listed it as one of their picks for the day.^65^

The BBC's Audience Research Department was the first to consider how viewers reacted to *TTN*. It surveyed a sample of viewers, as was typical for such productions, and these viewers rated the programme very highly: nearly a third of those surveyed (32 per cent) gave the programme an A +, while nearly half (48 per cent) gave it an A. Many viewers cited by the audience researchers stressed how ‘real’, how true to life the play's depiction of Christine's experience seemed to them, with several respondents even ‘confirming, from their own experience, the accuracy of the picture’. Alison Steadman's performance in particular was singled out as being especially convincing, though some viewers found Shepherd's junior doctor Pearce ‘rather too scruffy and informal to be entirely believable’. Even the stage set—constructed because the producers were unable to use an actual hospital—seemed real to viewers, as ‘“even down to the tatty old loos”’.^66^ Reviewers for the national papers offered much the same evaluation. The *Daily Telegraph*'s Sylvia Clayton, for instance, praised the play's ‘spiky honesty’ and the ‘quiet, natural key’ of Steadman's performance, and noted that the set was so realistic ‘you could almost smell the unmistakable hospital odour of ether and antiseptic and floor polish’.^67^

Should viewers want to know what the teleplay's author wanted them to take away from Christine's story, they could find out from a feature interview with Griffiths published in *Radio Times* the week *TTN* aired. ‘What angers [Griffiths],’ wrote his interviewer, the *Observer* theatre critic Robert Cushman, ‘is the profession's “extraordinary insensitivity to the wholeness of human beings”.’ Griffiths himself described Christine's experience as an exacerbated version of a systemic problem:

‘… If you've ever been in hospital you'll know how you immediately regress to infantilism … and that's for you [Cushman] and me—middle-class, professional, highly articulate, capable of aggression. What is it like for a woman with a potentially terminal condition who is used in precisely that way and without the resources, without the tradition of getting what you want that we have even if we don't exercise it?’^68^

Certainly many viewers seemed to agree with Griffiths's and Cushman's assessments. The *Radio Times* received a large number of letters in response to the programme and to Cushman's interview with Griffiths, although the magazine ran only excerpts from a few of the letters received. Where the headline on the *Radio Times* letters page offered two possible ways to regard *TTN*—‘True to life—or scary?’—the men and women who wrote in suggested a third option: true to life *and* scary. One such response was from a non-medical reader who claimed that Christine's story, though fictional, was far from exceptional:

… both medical men [in the *Tonight* programme after *TTN*] insisted that the kind of situation shown in the play could only be a very exceptional and a ‘one-off’ case. Nonsense! This kind of pompous behaviour is happening all the time, to judge from my own and many other people's experiences in hospitals. … Not unless and until the medical profession accept their patients as whole, knowledgeable, thinking human beings will they regain the respect and support of the public.^69^

The *Radio Times* also allowed producer Ann Scott to respond to critics, such as those doctors who felt the portrayal of their profession unfair, on the magazine's letters page. Scott confirmed that she and the production team had taken advice from several people with professional or personal experience of breast cancer treatment. In the end, she argued, the production team was satisfied that Christine's experiences ‘were not necessarily typical, but they were true’, and though fictional, she was neither an extreme nor isolated case.^70^

Elite critics, meanwhile, considered *TTN* both on its own merits and as one in a series of statements by a ‘political’ writer. The reviewers on the BBC Radio 3 programme *Critics' Forum* agreed that *TTN* was very well written, acted, presented and produced, and that it was, as poet Peter Porter put it, ‘very much the real thing’.^71^ Some of those on *Critics' Forum* argued that this ‘political’ play was too simplistic in its portrayal of the interplay of class politics and medical paternalism.^72^ But *Guardian* critic Peter Fiddick, while also praising the play, argued that by transmitting the discussion with medical authorities immediately afterwards, the BBC had undermined the power of Christine's story. The *Tonight* panel discussion that aired afterwards, he suggested, was intended to calm those upset by what the play said about the state of patient experience of medicine, the

… worried women [who] had been phoning the BBC, disturbed more than enough by the central idea that unless they, the putative patients, went in demanding their rights like subscribers to Hospital *Which?*, the same treatment could await.^73^

Fiddick understood why Griffiths and the play's producers had agreed to the panel discussion as a necessary compromise, even if it ‘de-fanged’ the drama's message somewhat. But he also drew attention to the fact that the credits indicated that the play was based on Griffiths's wife's diary. This, Fiddick pointed out, meant that medical professionals could not shrug off *TTN* as merely drama, ‘they could not face the author and charge: “It could not happen”.’^74^

But perhaps the most interesting effort to make sense of *TTN* came not from elite cultural critics, broadsheet reviewers, or the pages of the middlebrow *Radio Times*, but from everyday women who wrote in to the well-known tabloid ‘agony aunt’ Marje Proops. The Sunday after *TTN* aired, Proops asked her substantial *Sunday Mirror* readership ‘Did that play scare the life out of you?’^75^ In contrast to the BBC's high viewing figures, Proops offered her own straw poll of her friends and colleagues, noting that many of the women she knew avoided watching the play, fearful that it would be gruesome or worrisome; she also noted that many of those who did watch believed the play's depiction of doctors' ‘casual attitudes’ towards women would discourage women from seeking medical advice about breast lumps. Proops focused on what she described as the ‘scandalous’ incident of the consent form and what it implied about how hospital staff treated patients. She asked her readers:

I've been assured by hospitals, consultants, and nursing staffs that while indifferent treatment does sometimes happen, the majority of hospitals treat patients sympathetically.

*Hmm*, was my cynical reaction. I hear different stories. I'd like to hear yours.^76^

Proops received hundreds of replies, and two weeks later the *Sunday Mirror* ran a two-page feature headlined ‘The Fear that Grows from a Tiny Lump’.^77^ As the headline implies, although Proops had asked her correspondents to discuss their experiences of breast cancer treatment, some addressed their experience of breast cancer more broadly, not just their dealings with hospitals, doctors and nurses. Although Proops received what she termed a few ‘embittered’ letters, most respondents wrote that individual health professionals had been kind and careful in their treatment (‘He cared—I trusted’ read a typical subhead). Likewise, many of the excerpts from reader letters indicated that many women about to undergo surgery had had far more information about their condition and possible outcomes than Christine. The *Sunday Mirror* feature, for instance, quoted a Devon husband who wrote that ‘The surgeon has explained everything to my wife throughout and every kindness has been shown.’^78^

Even so, Proops noted a potentially contradictory attitude amongst the viewers who saw *TTN*. While the letters ran three to one ‘in praise of the skill, care and understanding [letter writers] were shown in hospital’, the majority of those letter writers also felt, as Proops put it, ‘strangely enough, that the BBC play was valid—even those who were full of praise for the hospitals also believed that the situation described was possible’.^79^ In other words, even amongst those who had had positive experiences of treatment, the majority believed that Christine's ostensibly fictional experience was entirely possible in real life. Several writers even reported that very similar events had happened to them. A Glamorgan woman wrote that she had been reassured that the consent form was ‘just a formality, and that her lump couldn't be anything serious’ but then awoke in the ward to find her breast gone.^80^ A woman writing from Aylesbury explained that

It was 10:30 p.m. when the doctor thrust the consent form at me and in the dim light I was unable to read a word of it. I started to ask a question, but he threw his hands in the air and said he couldn't possibly say anything about the operation, and I might have one breast off or even two.^81^

An East London reader wrote that after an initial biopsy and reassurance that all was fine, her surgeon reappeared later in the day,

smiled, and said ‘Sorry, my dear, we have in fact now decided that your lump is malignant and we are going to remove your breast.’ Before I could say anything he walked away. Never at any time did I receive any help coming to terms with this terrible loss.^82^

Despite condemning the attitudes and actions of those who behaved callously toward patients, Proops and those who wrote to her sustained a largely positive attitude towards breast cancer treatment generally—or at least about the treatment's ultimate medical value and their own abilities to eventually adjust to it. Griffiths had noted in the *Tonight* discussion aired after *TTN* that even if Christine's experience in hospital had been bad, she was better off for having received treatment. And *TTN* itself had reiterated the same message strongly: in his discussions with Christine, Dr Pearce made it clear that her medical treatment had ultimately been the best course of action, as well as skilfully performed by the distant Mr Staunton. Proops's respondents generally took the same position, feeling that even if their experiences as patients left much to be desired, they were still better off having had treatment than not. One Birmingham woman summed up this attitude: ‘Now I have no regrets. To every woman I say, go as soon as possible, have no fears at all. To lose a breast is better than losing your life.’^83^

This message, that breast cancer treatment was worthwhile even if the circumstances of its delivery needed improvement, appears to have been a hard sell. As discussed previously, the educational experts consulted before *TTN* was produced had worried that the play might hinder the cause of cancer education, but agreed that *TTN*'s broader dramatic value, together with the statement it made about the need for changes in medical institutions, outweighed these concerns. Not long afterward, however, survey research conducted by the BBC would suggest that the educators were right to worry, for *TTN*'s dramatic portrait of breast cancer treatment was apparently able to subvert the scientific ‘good news’ presented about cancer elsewhere on television. A report of the broadcaster's General Advisory Council had analysed the reception of a three-part factual programme broadcast in November 1975, *The Changing Face of Medicine*, portions of which focused on new thinking about the causes and cures of cancer. The BBC's audience research team were not only concerned to see how much these programmes had enlightened viewers as to specific ideas about cancer, but also how they had shaped viewers' attitudes towards cancer and its treatment.^84^ The airing of *TTN* in early December was deemed ‘unfortunate’, as those viewers being surveyed for their opinions about *The Changing Face of Medicine* received a questionnaire just after the play was broadcast, and the researchers fretted that ‘the play might overshadow any effects of [the documentary] series on viewers’ attitudes towards cancer or towards the medical profession'.^85^ Indeed, the drama did end up overshadowing the documentary in the minds of at least some viewers: the questionnaire showed that a greater percentage of those who viewed both *TTN* and *The Changing Face of Medicine* rated themselves as ‘more worried’ about cancer than those who had only seen the documentary. What's more, those who had viewed both *TTN* and the documentary also proved ‘less likely’ to believe the statement ‘I have a lot of faith in the medical profession’ than those who had only watched the documentary.^86^ There were, of course, multiple ways to make sense of these findings: perhaps those viewers already sceptical of the documentary's message were also more likely to watch *TTN*. But this was not how the BBC's audience research team interpreted the results. Instead, they believed this finding was evidence that a powerful drama, even with a discussion programme afterwards as damage control, had subverted the positive message promoted by a medical documentary.

What were *TTN*'s viewers, many of whom had found confirmation of their fears or even their own experiences in Christine's story, to do now that they had articulated these? Marje Proops and the *Sunday Mirror* had offered many women who had undergone breast cancer treatment (and even the men in their lives) a chance to say out loud, in public, that while their treatment for cancer may have succeeded, as patients they had been treated badly. But while Proops's column allowed those women to express their dissatisfaction, and allowed readers who felt similarly to see they were not alone, no concrete solutions were offered, only the examples of doctors and hospital staff who had been both kind and truthful. Nor did the *Sunday Mirror* (or any other of the venues where *TTN* was discussed) recommend that readers concerned about their treatment contact the Patients Association, the chief group working to make Britons aware of their rights in medical situations and pressing medical institutions to formally acknowledge their patients' individual autonomy.^87^

The only group offering a prescription for change offered the same one Griffiths had—speaking up—albeit with a slightly different rationale. In the small but growing women's health movement, feminist commentators found in Christine's story evidence of an inherently patriarchal nature of a health care system where women in particular had little control over their fates. For these commentators, the solution was obvious: women needed to act to re-establish that control, both individually and collectively. In her review for the feminist monthly *Spare Rib*, breast cancer patient Joan Scott noted that the play's Dr Pearce had used Hippocrates to argue that doctors needed to relate to patients as equals. But she also argued that women needed to act too, as Christine had, ‘to resist authoritarian or patronising attitudes if we encounter them’. This individual action was not simply in a woman's self interest, but part of a collective mandate:

It is not enough that when we are having surgery we are suffering and scared. We must also rally our wits and defend ourselves against the consequences of other peoples' incompetence, uncaring attitudes, or reluctance to face our needs. When we get out we can start/resume agitating for a better health service; but while ‘in’, if we want to take responsibility for ‘our bodies, ourselves’, we can't afford to be passive.^88^

Feminist academics also invoked *TTN* as an excellent illustration of the need for women to get, control, and share information about their health and illnesses. Social scientists Joyce Leeson and Judith Gray, in a pivotal 1978 text on women and medicine, wished the teleplay ‘could be shown to the staffs of all surgical units every few months!’ They (unlike most reviewers) noted that even as the play intended to show a young woman finding the strength to resist and demand in the face of medical authority, the predominant voice in the play was not Christine's but Pearce's. Nevertheless, Christine was lucky to find

a young house surgeon who is prepared to talk to her (although he does not seem keen to *listen* to her), and she is able to learn something about what has happened, and about what the future may hold in terms of life and death.^89^

But while Leeson and Gray approached *TTN* through the lens of gender politics, they agreed with Griffiths about the solution to the problems epitomised by Christine's experience. The best way to prevent such experiences in future was to act, individually and collectively, to regain power and agency for women within the health care system—in junior doctor Pearce's words, to demand.

## Conclusion

As a product of individual experience, as powerful (and political) art, and as a lightning rod for viewers' responses, *Through the Night* provided medical experts, educators, activists and everyday Britons with an opening to talk publicly about breast cancer treatment, and especially to consider the patient's experience of it. Thanks to an unusually rich array of sources documenting both the play's production and its reception, *Through the Night* also provides historians of British medicine with insight into those experiences and into the efforts to change them, as well as a glimpse of the complicated negotiations that conditioned media discussions of medical subjects in the 1970s. In this case, medical professionals and health educators hoping to change practices around cancer treatment threw their support behind the production of the play, as it provided them with more ammunition for their fight to improve patients' experience of that treatment. At the same time, the play provided critics from outside the system, such as feminist activists, with a compelling, realistic portrayal of the types of injustices they sought to correct. For its creator, *TTN* was another salvo—albeit an especially personal one—in a larger political project, borne out of a concern with the mechanics of class, authority and power in a changing Britain. And for many of the everyday Britons who watched and responded to it, this television play provided proof that their feelings of powerlessness in the face of medical authority were anything but groundless. Examining *TTN* and professional and public responses to it thus reveals the complexity that surrounded the emergence of the patient's ‘voice’ in 1970s Britain. Encouraged by professionals with their own agenda, this voice expressed its dissatisfaction with the way medical treatment was delivered. This was not a challenge to medicine's authority, but a call for doctors, nurses and hospitals to reform themselves and deliver care more humanely.

While *Through the Night* provided a prompt for professional and public discussion of the patient's experience of breast cancer treatment, that discussion revolved around how patients were treated by doctors, nurses and hospitals, rather than the treatments they received. The same year that *TTN* aired, US journalist Rose Kushner published a popular book marshalling medical evidence against American surgeons' continued reliance on the Halsted radical as well as the ‘one-step’ operation.^90^ But in Britain, specific critiques of the mastectomy and other cancer therapies and their effects on women's bodies emerged slightly later, in the late 1970s and early 1980s, when they would be articulated by British photographer Jo Spence and other feminists analysing breast cancer treatment and the gendered politics of embodiment. Nor was *Through the Night* intended as a feminist critique of health care relationships or institutions, even if feminists would employ it that way. The play and those who commented on it largely regarded Christine's experience not as a woman's problem, but a problem that was particularly likely to happen to women. This was a political critique that could be read as sympathetic to feminist critiques of medical institutions, but that was not originally framed as such. Nevertheless, hinting at what was to come, the breast cancer patients who responded to the play, and to calls like Proops's to make sense of it, spoke compellingly of their experiences as patients and their anger at institutions and circumstances that deprived them of control over their own bodies, even if a feminist vocabulary was not yet available to them.

## Funding

The research for this article was conducted as part of the Constructing Cancers project, Wellcome Trust Programme Grant 068397.
